# From the Cochrane Library: Hydrosurgical Debridement Versus Conventional Surgical Debridement for Acute Partial-Thickness Burns

**DOI:** 10.2196/37030

**Published:** 2022-05-04

**Authors:** Ryan E Kokoska, Mindy D Szeto, Torunn E Sivesind, Robert P Dellavalle, Justin C R Wormald

**Affiliations:** 1 School of Medicine Indiana University Indianapolis, IN United States; 2 Department of Dermatology University of Colorado Anschutz Medical Campus Aurora, CO United States; 3 Nuffield Department of Orthopaedics, Rheumatology and Musculoskeletal Sciences University of Oxford Oxford United Kingdom

**Keywords:** Cochrane, systematic review, randomized controlled trial, hydrosurgery, hydrosurgical debridement, debridement, burns, wound healing

Partial-thickness burns often require surgical excision with dressings or reconstruction. Standard of care includes early debridement (tangential excision of nonviable tissue) followed by split-thickness skin grafting. The goal of debridement is to reach a plane of viable tissue, while sparing healthy, uninjured tissue, expediting healing and minimizing scarring. Conventional debridement (scalpel or knife) is potentially limited by inaccurate differentiation between viable and nonviable tissues, with resultant delayed healing and greater scarring. Hydrosurgery is an alternative tool for surgical debridement that uses pressurized saline and a vacuum system to create a Venturi effect, ideally improving debridement accuracy and tissue-sparing. The Cochrane systematic review “Hydrosurgical debridement versus conventional surgical debridement for acute partial-thickness burns” analyzed existing randomized controlled trials (RCTs) enrolling participants with acute partial-thickness burn injuries requiring debridement and grafting; this yielded one eligible study randomizing 61 pediatric patients to either conventional debridement (n=31) or hydrosurgery (n=30) [1].

In this RCT, no clear differences were observed in the mean time to complete healing (mean difference [MD] 0 days, 95% CI –6.25 to 6.25), postoperative infection risk (risk ratio 1.33, 95% CI 0.57-3.11), operative time (MD 0.2 minutes, 95% CI –12.2 to 12.6), or 6-month scar outcome (MD not computed). Study conclusions were very low certainty on the GRADE (Grading of Recommendations Assessment, Development and Evaluation) assessment, showed a high risk of reporting bias, and were limited by the small sample size (not powered to detect differences in primary outcomes). Generalizability was limited, as the study focused on a pediatric population and smaller burn injuries (3%-4% of total body surface area). No information was reported on clinical resource use, health-related quality of life, or adverse events. The authors concluded that it remains unknown if hydrosurgery is superior to conventional surgery for treatment of middepth burns.

Following the publication of the Cochrane review, no further RCTs have been published that compare the efficacy of hydrosurgical debridement to conventional blade debridement for burns. However, one study is still “awaiting classification,” and one multicenter RCT (n=137) is underway to examine long-term (12 months) scar quality for hydrosurgical versus conventional debridement of dermal burns [2].

In addition to its application for burns, there is evidence for hydrosurgery treating other dermatological pathologies. For example, in a study of axillary osmidrosis (n=93), hydrosurgery showed improved patient satisfaction and fewer postoperative complications compared to traditional surgery [3]. Case reports of severe phymatous rosacea, which currently lacks standard surgical guidelines, document successful treatment with hydrosurgery [4]. Additionally, hydrosurgery can safely and rapidly debride various ulcer types in outpatient settings [5]. As the COVID-19 pandemic continues to decrease the availability of inpatient rooms and services, the possibility of providing outpatient hydrosurgical debridement for wounds may be important for continuing patient care. Dermatologists manage numerous wounds in daily practice; therefore, providers should be informed of the current recommendations for wound debridement. Future research should include additional high-quality RCTs comparing the efficacy of hydrosurgery versus standard debridement for burns. Outcome measures could focus on patient-reported scarring and adverse events. This would increase the certainty and generalizability of the results, and provide evidence for procedural recommendations.

## Abbreviations

GRADE: Grading of Recommendations Assessment, Development and Evaluation
JID: Journal of Investigative Dermatology
MD: mean difference
NIHR: National Institute of Health Research
RCT: randomized controlled trial
